# Directed Evolution and *In Silico* Analysis of Reaction Centre Proteins Reveal Molecular Signatures of Photosynthesis Adaptation to Radiation Pressure

**DOI:** 10.1371/journal.pone.0016216

**Published:** 2011-01-13

**Authors:** Giuseppina Rea, Maya Lambreva, Fabio Polticelli, Ivo Bertalan, Amina Antonacci, Sandro Pastorelli, Mario Damasso, Udo Johanningmeier, Maria Teresa Giardi

**Affiliations:** 1 Institute of Crystallography, National Research Council, Monterotondo, Italy; 2 Department of Biology, University Roma Tre, Rome, Italy; 3 Martin-Luther-University, Plant Physiology Institute, Halle (Saale), Germany; Cinvestav, Mexico

## Abstract

Evolutionary mechanisms adopted by the photosynthetic apparatus to modifications in the Earth's atmosphere on a geological time-scale remain a focus of intense research. The photosynthetic machinery has had to cope with continuously changing environmental conditions and particularly with the complex ionizing radiation emitted by solar flares. The photosynthetic D1 protein, being the site of electron tunneling-mediated charge separation and solar energy transduction, is a hot spot for the generation of radiation-induced radical injuries. We explored the possibility to produce D1 variants tolerant to ionizing radiation in *Chlamydomonas reinhardtii* and clarified the effect of radiation-induced oxidative damage on the photosynthetic proteins evolution. In vitro directed evolution strategies targeted at the D1 protein were adopted to create libraries of chlamydomonas random mutants, subsequently selected by exposures to radical-generating proton or neutron sources. The common trend observed in the D1 aminoacidic substitutions was the replacement of less polar by more polar amino acids. The applied selection pressure forced replacement of residues more sensitive to oxidative damage with less sensitive ones, suggesting that ionizing radiation may have been one of the driving forces in the evolution of the eukaryotic photosynthetic apparatus. A set of the identified aminoacidic substitutions, close to the secondary plastoquinone binding niche and oxygen evolving complex, were introduced by site-directed mutagenesis in un-transformed strains, and their sensitivity to free radicals attack analyzed. Mutants displayed reduced electron transport efficiency in physiological conditions, and increased photosynthetic performance stability and oxygen evolution capacity in stressful high-light conditions. Finally, comparative *in silico* analyses of D1 aminoacidic sequences of organisms differently located in the evolution chain, revealed a higher ratio of residues more sensitive to oxidative damage in the eukaryotic/cyanobacterial proteins compared to their bacterial orthologs. These results led us to hypothesize an archaean atmosphere less challenging in terms of ionizing radiation than the present one.

## Introduction

The space environment of the solar system is a highly dynamic milieu distinguished by the presence of high energy particles and ionizing radiation potentially hazardous for all living systems. Natural radiation consists of electrons and protons trapped by planetary magnetic fields, protons and heavy nuclei produced in energetic solar events, and cosmic rays produced in supernova explosions inside and outside our galaxy. The primary cosmic beam, composed of very energetic protons and heavy atomic nuclei, is partially converted into secondary neutrons by collisions with matter; in turn these neutrons can produce additional radiation types of various energies [1], [2]. Near Earth radiation composition and specification is central for the habitability of environments within which life has developed during the early phases of formation of solar and planetary systems [3].

Throughout the geologic eras photosynthesis was likely adjusted to the presence of the ionizing radiation coming from space [4]. The earliest photosynthetic organism was a bacterium perhaps activated by hydrogen sulfide, with metabolic capabilities similar to those of modern Cyanobacteria [5]. The evolution of aerobic photosynthesis set in motion the development of an oxygen-rich atmosphere that dramatically transformed the chemistry of the Earth, imposing new challenges to evolving organisms also in terms of radiation-induced damage.

Among the three domains of life on Earth, only Eukarya and Bacteria are capable of using solar energy to grow, with several differences in the energy conversion. Eukarya perform oxygenic photosynthesis using water as a source of electrons. For this purpose, they are equipped with two photosystems (PSs) PSII and PSI, acting in series and creating a light-driven flux of electrons from the high redox potential couple H_2_O/O_2_, to the low redox potential couple NADPH/NADP^+^. The electron flux is coupled to the generation of a proton gradient that drives the synthesis of ATP, ultimately leading to CO_2_ fixation [6].

Bacteria are capable of undertaking oxygenic or anoxygenic photosynthesis. Cyanobacteria are competent at extracting electrons from water and performing oxygenic photosynthesis. Like Eukarya, they possess two PSs, but lack the compartmentalization of the processes in specialized organelles. Phylogenetic analyses indicate that Cyanobacteria are closely related to plant and algal chloroplasts, which are the organelles that house the PSs in eukaryotic cells [7], [8]. All other bacteria use only one photosystem and, for thermodynamic reasons, they cannot utilize water, but use compounds such as H_2_S as electron donors; these organisms are competent at autotrophic growth.

PSs are macromolecular protein-chlorophyll assemblies composed of a reaction centre (RC), driving photochemical charge separation and electron transport, and inner and outer antennae carrying out light energy absorption, dissipation and transduction [9], [10].

The organization of the Eukarya PSII RC is comparable to that found in purple photosynthetic bacteria, but with the addition of the oxygen-evolving complex. This RC type contains two membrane polypeptides (L and M in purple bacteria, and D1 and D2 in eukaryotic organisms), each containing five transmembrane helices, hosting the electron transport carriers and cofactors [11], [12]. The PSII D1/D2 heterodimer has a crucial role in the overall photosynthetic process and plant performance. A characteristic feature of the PSII reaction centre heterodimer is the rapid, light-dependent catabolism of the D1 protein, which is one of the main sites of damage caused by a wide variety of environmental factors [13]–[16]. The D1 protein is the product of the evolutionarily very conserved *psb*A gene, which has been found in genomes of plastids, cyanobacteria, and cyanophages [17]. It has been hypothesized that the co-opting of this gene into the virus genome provides a significant advantage for the fitness, reproduction and propagation of certain cyanophage types [18]. In Cyanobacteria, the relevance of this protein is also highlighted by the occurrence of a small gene family, whose members encode D1 isoforms slightly differently in their amino acid composition, activated in different stress conditions. On the contrary, in higher plants and microalgae, a single *psb*A gene has been identified, but despite these differences plants and cyanobacteria have adopted very similar *psb*A gene expression mechanisms [19]. At post-transduction level, the D1 reversible phosphorylation has been proposed as a more energy-efficient mechanism selected during evolution to substitute the function of multiple gene members [16]. The D1 rapid turn-over, the expression of different D1 isoforms in different light conditions [20] and the induction of a “silent” D1 isoform in microaerobiosis [19] indicate not only the relevance of D1 in maintaining a functional PSII, but also the significance of its aminoacidic composition to rapidly respond to environmental changes, that could result from a high level of evolutionary selection pressure.

The molecular architecture of the RC at atomic level is known for the purple photosynthetic bacteria *Rhodobacter sphaeroides* and the thermophilic cyanobacterium *Synechococcus elongatus* [21]–[23].

In this work, we adopted an *in vitro* directed evolution strategy targeted at the D1 protein to create ionizing radiation tolerant chlamydomonas strains, exploiting as evolution selection pressure proton or neutron sources capable of generating radicals. The reduced radical sensitivity of the selected strains was proved on the ex novo produced site-directed mutants by the stability of their photosynthetic performance under high fluency rates. By this approach we identified mutants tolerant to free radicals and the corresponding D1 aminoacidic substitutions which render the protein less prone to oxidative damage compared to the wild type. An *in silico* analysis of the L/D1 amino acid sequences of Bacteria, Cyanobacteria and Eukarya suggested that radiation-induced oxidative damage could had been one of the driving forces in the evolution of L/D1 proteins; this approach also gave evidence of a molecular signature of different levels of ionizing radiation on Earth during the evolution of the photosynthetic apparatus.

## Results

### Selection of *Chlamydomonas reinhardtii* strains tolerant to ionizing radiation

A directed evolution strategy was exploited to produce chlamydomonas mutant libraries with improved tolerance to space ionizing radiation. Random mutagenesis was targeted at *psb*A gene encoding D1 to produce proteins with novel properties. This has been achieved by error-prone PCR mutagenesis through an iterative process consisting of a recombinant generation to create combinatorial libraries. The resulting pool of *psb*A fragments was delivered into the deletion mutant, Del1, of *C. reinhardtii* [24] by particle gun bombardments. This mutant has a defined deletion in the chloroplast-encoded *psb*A gene and is unable to grow photoautotrophically, as it cannot produce a functional D1 protein. Acetate is needed as carbon source as minimal media does not support its growth. The successful integration of the modified *psb*A fragments into the Del1 chloroplast genome restores the expression of functional D1 protein and cell photoautotrophic growth. Thus, minimal media are used to select the photosynthetically active colonies.

In order to isolate transformants, not only competent for photoautotrophic growth, but also tolerant to ionizing radiation, a pool of mutants was exposed to high energy neutrons, neutrons, neutrons plus high light and protons. Neutron and proton energies utilized for the selection were similar to those occurring in space [2]. About 2000 transformed cells were subjected to the ionizing radiation treatments, and 32 colonies overcoming the radiation-induced stress were analyzed by *psb*A sequencing. Among them, twenty strains were identified as different mutants hosting both single and double mutations (Table S1). The analysis of the *psb*A nucleotide sequence of these strains revealed that mutations were mainly located in two structural regions of the D1 protein. The first region is the D1 Q_B_ binding pocket toward the C-terminus which comprises part of the IV and V α helices and part of the large extrinsic loop towards the stromal side of the thylakoid membrane. Mutations in this region may affect the binding of the quinone electron acceptor and the D1 protein turnover [19], [25]–[28]. No mutants hosting aminoacidic substitutions in the Q_B_ pocket residues directly involved in plastoquinone binding, or binding stabilization were identified. The second region comprises the D1 amino acids close to the redox-active tyrosine 161 (TyrZ), on the lumenal side expanding to the oxygen evolving complex (OEC) [21], [29]. The oxidized TyrZ residue is essential in electron transfer, since it mediates the extraction of an electron from the cluster of four manganese ions of the OEC that binds substrate water [6]. Strains harboring mutations in both protein regions have been also identified. Analysis of the amino acid changes characterizing the selected mutants highlights a clear trend towards the replacement of residues highly prone to oxidative damage, such as aromatic and aliphatic residues, with residues less prone, such as shorter chain aliphatic residues and polar residues (Table S1).

### Production and characterization of chlamydomonas D1 site-directed mutants

**Figure 1:**
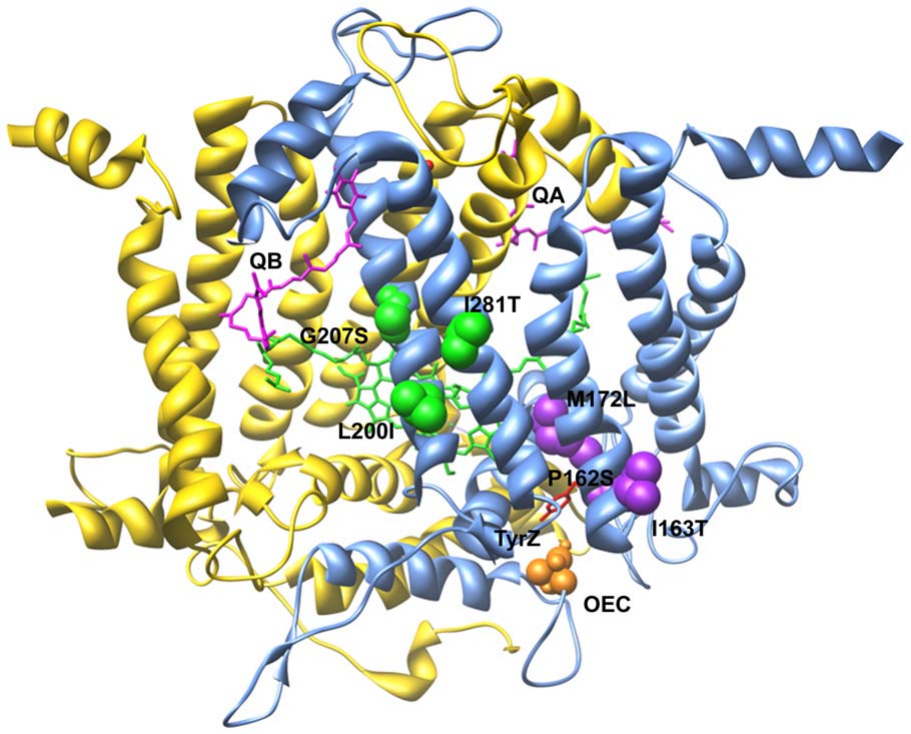
Schematic representation of chlamydomonas D1-D2 heterodimer showing the positions of the site-directed D1 aminoacidic substitutions. Positions of the modified amino acids in the D1 proteins produced by site-directed mutagenesis are viewed in the context of 3D PSII structure. The overall structure of D1 and D2 proteins of PSII and cofactors involved in electron transfer Q_A_, Q_B_, non-heme iron, P680 and OEC, is presented in a view perpendicular to the membrane plane according to [29], [21]. The D1 protein is in light blue and D2 in yellow. The quinone Q_A_, bound to D2, and the quinone Q_B_, bound to D1 protein, are represented in magenta; the non-heme iron in red; the chlorophyll dimer P680 in green. The TyrZ is displayed in sticks and coloured in red and, below, the Mn cluster, the Ca^2+^ ion and the oxygen atoms of the OEC are shown as orange spheres. The D1 amino acids which were substituted are displayed in space fill representation and coloured in green (for mutations clustering on the IV and V α helices) and purple (for mutations close to the redox-active TyrZ and OEC).

**Figure 2:**
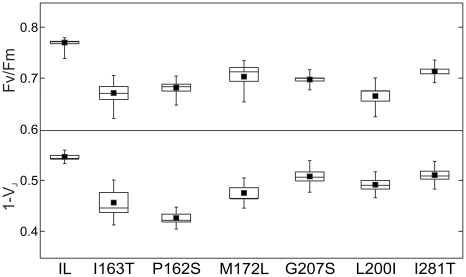
Maximum quantum yield and electron transport efficiency of the site-directed chlamydomonas D1 mutants. The mutants show a generally lower maximum quantum yield of PSII photochemistry (Fv/Fm) and a reduced efficiency of the electron transport through PSII primary and secondary electron acceptors (1-V_J_) compared to the control strain, IL. Fluorescence parameters of the reference strain, IL, and the D1 site-directed mutants of *C. reinhardtii* were calculated from the fluorescence induction curve. The statistical distributions for the maximum quantum yield of PSII photochemistry, Fv/Fm  =  (Fm-Fo)/Fm, and the efficiency of the electron transport between the primary (Q_A_) and secondary (Q_B_) PSII electron acceptors, 1-V_J_  = 1 - (F_2ms_-F_o_)/(Fm-F_o_), are presented as box and whiskers plots. The measurements were performed on liquid cell cultures containing equal amounts of chlorophyll (14±1 µg ml^−1^) after 10 min of dark adaptation. The statistical analysis was performed on average values, obtained from at least four experiments, n = 9–20. The mutants resulted statistically different from the reference strain at P = 0.05.

The reported results revealed that single aminoacidic substitution in the D1 protein could contribute to ionizing radiation tolerance. To exclude the possibility that additional random mutations induced by the radiation exposure could confer the observed tolerance, a set of the identified amino acid substitutions were introduced by site-directed mutagenesis in untransformed strains (Table 1). The choice of the aminoacidic substitutions was made to include mutations located in two different structural regions of the D1 protein, near Tyr161 and the OEC (I163T, P162S, M172L) and near the Q_B_ binding pocket (G207S, L200I, I281T) (Fig. 1). Physiological characterization of the obtained mutants was carried out by estimating the growth parameters and the photosynthetic efficiency. The mixotrophic growth rate was followed for a period of four days, revealing very similar trends among the different mutants and compared to the control reference strain IL (Fig. S1A). In contrast, the mutants and the control strain showed differences in their chlorophyll content (Fig. S1B) and photosynthetic performance. The mutants generally showed a lower maximal quantum yield of PSII photochemistry (Fv/Fm) and a reduced efficiency of the electron transport through PSII primary and secondary electron acceptors (1-V_J_) compared to the control strain (Fig. 2). These findings clearly indicate a reduction in the efficiency of the light energy utilization in the mutant strains compared to control in physiological conditions. Thus, these statistically significant differences indicate the relevance of a single amino acid substitution in D1 for the function of PSII, in agreement with previous results (Rea G, unpublished data). The extent of the reduction in the fluorescence parameters was not strictly related to the position of the amino acid substitution, as a general reduction of PSII performance was observed in both groups of mutants (Fig. 2).

**Table 1:** Description of amino acid substitutions in de novo produced site-directed chlamydomonas D1 mutants.

Mutants	Amino acid substitutions		Amino acid properties^a^hydropathy index/reactivity class/side chain polarity				Localization of the mutation in the protein
	wild type → mutated		wild type → mutated				
P162S	proline	serine	−1.6 (III)	nonpolar	−0.8 (0)	polar	near to Tyr_161_
I163T	isoleucine	threonine	4.5 (IV)	nonpolar	−0.7 (0)	polar	near to Tyr_161_
M172L	methionine	leucine	1.9 (V)	nonpolar	3.8 (IV)	nonpolar	near to OEC
G207S	glycine	serine	−0.4 (I)	nonpolar	−0.8 (0)	polar	in the helix IV of D1
L200I	leucine	isoleucine	3.8 (IV)	nonpolar	4.5 (IV)	nonpolar	in the helix IV of D1
I281T	isoleucine	threonine	4.5 (IV)	nonpolar	−0.7 (0)	polar	in the helix V of D1

The reported set of D1 mutations could account for the radiation tolerance previously found in D1 random mutants surviving neutron and proton exposure, and was selected to produce the corresponding site-directed mutants.

a The side chain polarity is presented according to [53], the hydropathy index is cited as in [54], the amino acids reactivity classes are reported in Table 2.

Analysis of the three-dimensional structure of PSII indicates that the mutants are not expected to cause gross structural rearrangements either. In fact, Ile163 and Ile281 face lipid molecules without establishing a direct contact with them (approx. 4 Å distance in both cases). Thus mutation to Thr is expected to be easily compensated by a rearrangement of the lipids conformation. Pro162 packs against Phe168 and modeling of mutation to Ser does not evidence significant packing differences while an additional hydrogen bond to Asp170 backbone carbonyl oxygen is predicted to be established. G207 is located on the surface of the solvent accessible Q_A_ entry channel and thus mutation to Ser should be easily accommodated also in this case.

**Figure 3:**
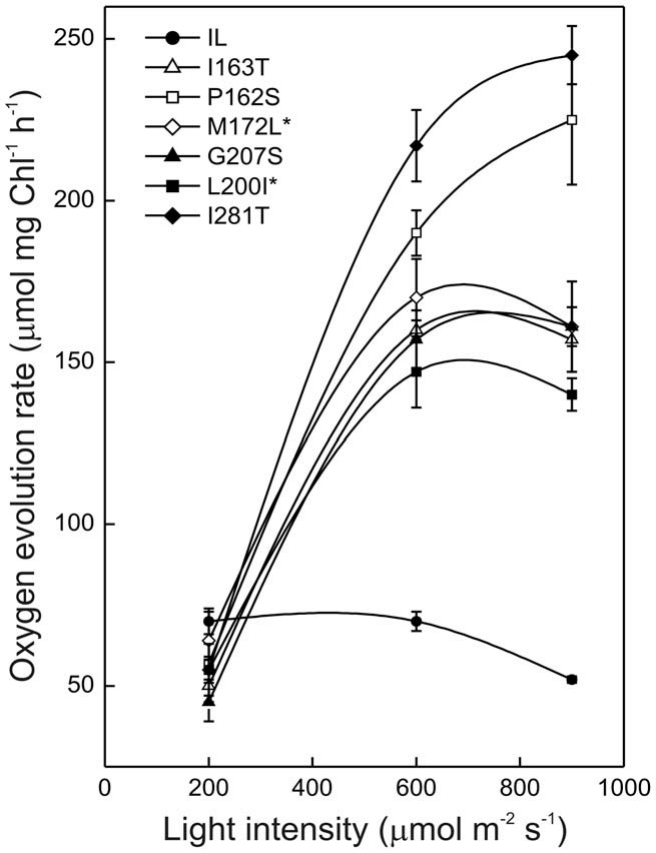
Light-dependent oxygen evolution capacity of the site-directed chlamydomonas D1 mutants. A higher oxygen evolution capacity under high photon fluency conditions was demonstrated in all the mutants compared to the reference strain, IL. The curves were recorded by gradual increase of the light intensity from 200 up to 900 µmol m^−2^ s^−1^. Measurements were performed on liquid cell cultures containing equal amounts of chlorophyll (15±2 µg ml^−1^), in the presence of 10 mM NaHCO_3_ as additional carbon source. Values are the average of three independent experiments, ±SE, n = 9. The mutants resulted statistically different from the reference strain at P = 0.05, except the cases M172L and L200I at 200 µmol m^−2^ s^−1^ indicated by an asterisk.

Despite the lower electron transfer efficiency, a higher oxygen evolution capacity under high photon fluency conditions was demonstrated in all the mutants compared to the reference strain, IL (Fig. 3). The rate of O_2_ production of the mutants and the control strain were very similar under 200 µmol.m^−2^ s^−1^ light intensity, which was already saturating for the photosynthetic reactions of IL strain. On the contrary, D1 mutants were able to achieve and maintain more than two-fold higher O_2_ evolution rate even under the very high light intensity of 900 µmol m^−2^ s^−1^.

### Photosynthetic efficiency of site-directed mutants

**Figure 4:**
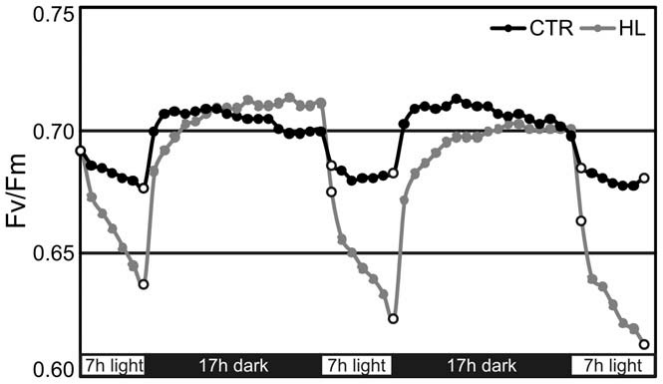
Daily trend of PSII photochemistry during two consecutive light/dark cycles in the reference strain IL. Cell cultures containing equal amounts of chlorophyll were immobilized on TAP agar medium and the Fv/Fm ratio was measured hourly in both growth (50 µmol m^−2^ s^−1^, CTR, black line) and high light conditions (150 µmol m^−2^ s^−1^, HL, grey line). Photoinhibition is evident by the reduction of the Fv/Fm recorded during the light phases under HL compared to CTR photon fluency. The open circles indicate the first and the last point of the light phase. The reported curves are representative of three independent experiments, n = 6. SE <3%.

**Figure 5:**
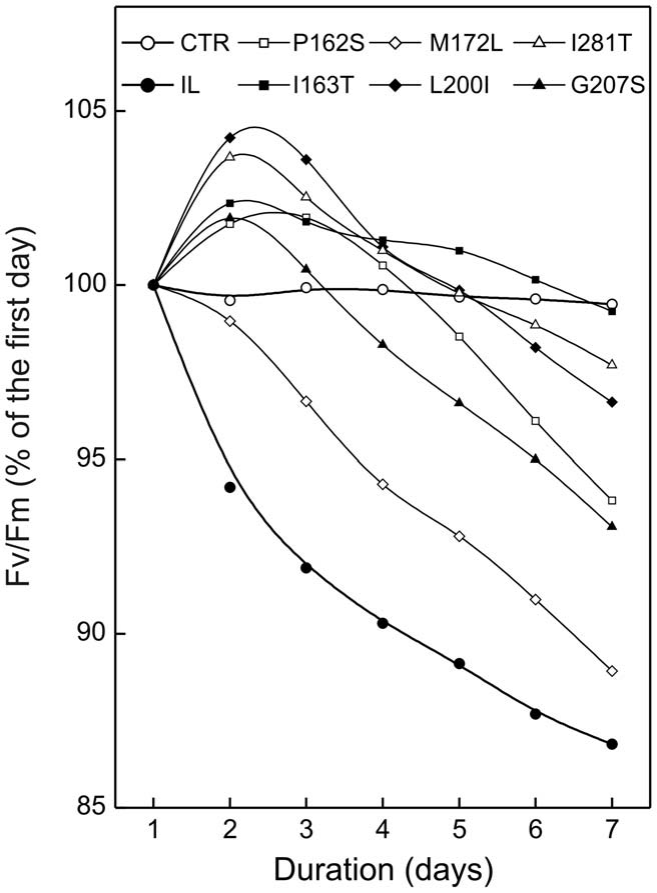
Time-course of PSII photochemistry at high photon fluency in the site-directed chlamydomonas D1 mutants. Cell cultures containing equal amounts of chlorophyll (80 µg) were immobilized on TAP agar medium and the Fv/Fm ratio was measured hourly in both growth (50 µmol m^−2^ s^−1^) and high light conditions (150 µmol m^−2^ s^−1^). The reported values are measured at the onset of each light phase and are reported as percentage of the Fv/Fm ratio calculated with respect to the first day. All the analysed strains displayed a good capacity to tolerate the applied photoinhibitory light intensities (150 µmol m^−2^ s^−1^). The maximal photosynthetic efficiency reduction was observed in the reference strain IL compared to the produced site-directed mutants. The control line (CTR) corresponds to an average value of all characterised strains and was obtained from samples exposed to 50 µmol m^−2^ s^−1^. The reported values are the average of three independent experiments; for the sake of clarity bars of standard errors are omitted, the maximum SE being <3%. The mutants resulted statistically different from the reference strain at P = 0.05.

Light intensity is a vital factor in photosynthetic organisms. However excessive doses can severely damage the photosynthetic apparatus due to production of high levels of free radicals. To test the capability of the selected mutants to cope with the challenge of the high irradiance conditions, we monitored changes in their PSII efficiency in the presence of stressful high light intensity by real-time measurements of the Fv/Fm ratio. In the reference strain IL under control light conditions, the changes of the Fv/Fm values as a result of two contiguous exposures to light and darkness were negligible (Fig. 4). On the contrary, under high irradiance, the PSII performance tended to decrease with the onset of the light, showing the pattern of accumulation of the photo-induced oxidative pressure. However, the reduction was reversible, since during the following dark phase a recovery of the maximum quantum yield of PSII photochemical reaction occurred (Fig. 4). We have to point out that the light intensity that the chlamydomonas strains could support depends strongly on the growth illumination and the physical phase of the nutrition medium. According to our experiments and in confirmation of the data presented in Fig. 4, when the algal cells are immobilized on a solid medium a light intensity as low as 150 µmol.m^−2^ s^−1^ could induce photoinhibitory PSII damage. The injury could be permanent, leading to the cell death, or reversible, depending on the stress duration. All the analyzed strains displayed a high capacity to tolerate the applied stress. In fact, over the monitored period, decrease of photosynthesis efficiency ranged from 1 to 13% (Fig. 5). The maximum reduction level was observed in the reference strain IL (13%), that showed the worst performance compared to the mutants. In this strain, in fact, after only two days of treatment, the maximum quantum yield dropped by 6%, and continuously declined during the subsequent days. Concerning the mutants, no correlation was observed between amino acid localization and photosynthetic activity. However, different mutants behaved in a different manner: I163T, L200I and I281T maintained very stable photosynthetic efficiency, reporting maximum 3.4% reduction of Fv/Fm values; G207S and P162S were less stable, gradually loosing about 7% of the initial activity. M172L provided results similar to IL, displaying a photosynthetic reduction of about 10%.

### Compositional analysis of bacterial, cyanobacterial and eukaryotic L/D1 proteins

**Figure 6:**
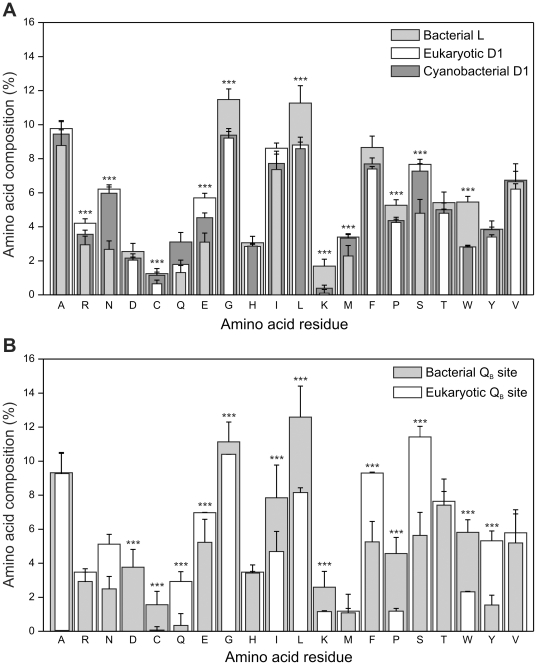
Comparison of the aminoacidic composition of *R. spheroides* L and *C. reinhardtii* D1 protein homologues. A) Comparison of the amino acid composition of *R. spheroides* L protein homologues (light gray columns), eukaryotic *C. reinhardtii* D1 protein homologues (white columns) and cyanobacterial *C. reinhardtii* D1 protein homologues (dark gray columns). Black bars indicate standard deviation values. B) Comparison of the amino acid composition of the Q_B_ binding region of *R. spheroides* L protein homologues (light gray columns) and *C. reinhardtii* D1 protein homologues (white columns). Black bars indicate standard deviation values. The asterisks indicate statistically significant differences between the Bacteria and Eukarya at p = 0.001.

The observation that the strains selected under ionizing radiation displayed amino acid changes consistent with tolerance to radiation-induced oxidative damage prompted a comparative study of the D1 amino acid composition. Bacterial, cyanobacterial and eukaryotic L/D1 proteins were analyzed to uncover the molecular signatures of the PSII complex adaptation to the radiation-induced damage occurring during the evolution of photosynthesis. Using *R. sphaeroides* L and *C. reinhardtii* D1 proteins as baits, homologues were retrieved from the NCBI sequence database and their amino acid composition was determined and compared (Fig. 6A). Although these proteins are highly conserved, analysis of the data reveals that the content of aliphatic and aromatic residues is significantly higher in Bacteria. In fact, aliphatic and aromatic residues represent almost 70% of the total residues in bacterial RC L proteins, while in eukarya D1 proteins the same residues represent less than 60% of the total. In particular, the percentage of Trp residues in bacterial L proteins is almost twice than that in eukaryotic D1 proteins, while smaller but still significant differences were observed in other residues (such as Gly and Leu) highly prone to damage by reactive oxygen species. Not surprisingly, the amino acid composition of cyanobacterial D1 proteins, in line with the evolutionary conservation of D1 proteins, is practically identical to that of the eukaryotic ones (Fig. 6A).

To investigate if conservation of residues building up the Q_B_ binding niche followed a different pattern, the same analysis was carried out on the sequence regions encompassing the quinone binding site, using as baits the 199–292 region of *C. reinhardtii* D1 protein and the ortholog 174–249 region of *R. sphaeroides* L protein. In addition, superimposition of the tridimensional structures has been carried out, revealing a Q_B_ binding niche backbone fold highly compatible in the 190–291 regions in which the aminoacidic sequences share approx 25% identity (Fig. S2). The comparative compositional analyses highlight a complex pattern showing a significantly higher content of the highly prone to oxidative damage Ile, Leu, Trp and Cys residues in bacterial proteins compared to eukaryotic ones, whereas only Phe and Tyr follow an inverse trend (Fig. 6B).

## Discussion

The evolution of photosynthetic organisms and the concomitant release of oxygen into the atmosphere caused a tremendous environmental change of our planet. Before oxygen release into the atmosphere, there was no ozone layer to screen ionizing radiation, thus it can be hypothesized that photosynthetic bacteria were exposed to high energy radiation from space. Radiation induced decomposition of water generates several reactive species (e.g., H_2_O_2_, H_2_, OH**^•^**, HO**^•^**_2_, e^−^_aq_, H^+^) which, in the presence of oxygen react further, so that OH**^•^** and O_2_^−^ are essentially the only species present in oxygenated water solutions [30]–[32]. OH**^•^** is known to be far more reactive than O_2_^−^, therefore the major fraction of OH**^•^** is removed by the most reactive sites. In proteins, aromatic, sulfur-containing and aliphatic amino acid side chains are known to be the main targets of OH**^•^**-mediated damage. Aliphatic amino acids become more reactive with increasing number of methyl groups but the reaction with aromatic amino acids is faster and easier for addition of OH**^•^** to double bonds, with Trp residues being the most prone to oxidative damage by reactive oxygen species [30]–[32].

In line with these considerations, we isolated chlamydomonas strains tolerant to ionizing radiation by exposures of different D1 random mutant libraries to radical-generating proton or neutron sources with energies similar to those occurring in space. Sequence analyses of *psb*A gene in mutants overcoming the irradiation identified the replacement of deduced aliphatic and aromatic amino acids with more polar residues. Thus, the mutant's tolerance to radical-induced damage seems to be related to the substitution of residues highly prone to oxidative damage with residues less prone (Table 1; Table S1).

In order to prove this hypothesis, we evaluated the role of the identified D1 amino acid substitutions in *de novo* synthesized site-directed mutants, whose photosynthetic performance was characterized in both physiological and radical-generating stress conditions. The production of site-directed mutants allowed to exclude the presence of casual gene modifications induced by the radiation exposures, which could account for the observed tolerance in the D1 random mutants. The mutations led to an increased photosynthetic performance stability and oxygen evolution capacity in stressful light conditions (Fig. 3; Fig. 5). During photosynthesis, the excess light energy cannot be used for water oxidation and may deactivate photosynthetic electron transport and cause oxidative damage. Direct observation of reactive oxygen species by spin trapping electron paramagnetic resonance spectroscopy, demonstrated that excessive light can induce oxidative stress [33]–[35]. In particular, the interaction between free electrons with oxygen molecules, tyrosine residues and chlorophylls leads to the formation of dangerous radical species as singlet oxygen, hydrogen peroxide, hydroxyl radical, Tyr radical and chlorophyll triplets that can compromise the PSII functionality [16], [36]–[39]. Thus, our experimental results demonstrate that the D1 amino acid substitutions responsible for the radiation tolerance in the random mutants, could also account for the improved resistance under high light exposure of the site-directed mutants.

In the context of the evolution of the photosynthetic apparatus, as a kind of adaptation signature to the harsh and radiation-permeated archaean atmosphere, a lower relative content of highly prone to radiation-induced oxidative damage amino acids would be expected in bacterial RC proteins compared to the eukaryotic ones. Surprisingly, bacterial L proteins display a higher percentage of aromatic and aliphatic residues (Fig. 6). In this respect, it is particularly striking that bacterial L proteins have a Trp content almost double that observed in cyanobacterial/eukaryotic D1 proteins, the difference being even greater if only the Q_B_ binding region is analyzed. In fact, Trp is known to be an amino acid highly prone to radiation-induced damage [30], [31].

However, additional evidence supports our hypothesis. It is well known that many cyanobacterial viruses encode host-like photosynthesis proteins and most of them contain the *psb*A gene in their genome [18]. Interestingly, most of the cyanophages-encoded D1 proteins display the deletion of the first 58 to 94 amino acids, a region which, in the eukaryotic and cyanobacterial D1 proteins, is extremely rich in oxidative damage prone residues. As an example, the region 1–94 of *C. reinhardtii* D1 protein displays approx. 50% of aliphatic residues, 12% of aromatic residues and 4% of sulfur containing residues, which makes up to two thirds of the total residues possible targets of oxidative damage.

Furthermore, some studies point to a consistently less challenging archaean atmosphere even in the absence of the ozone layer. In particular, some authors speculate that archaean bacteria could be protected from radiation damage by a series of chemical and physical factors [40]. Among the chemical factors, CO_2_, sulfur compounds deriving from SO_2_ and H_2_S decomposition and CH_4_-generated hydrocarbon smog would have reduced UV flux to values similar to exposed present-day Earth [41]. Physical factors may have included lithic habitat protection against UV radiation and bacteria matting habits. In the latter case, a layer of dead organisms would have protected organisms underneath by compounds functioning as UV shields [41]. Biochemical protection towards UV radiation is in fact postulated to have evolved in the early Earth with the production of compounds such as mycosporine-like amino acids, scytonemin, and carotenoids which would have provided biological protection against the complete UV range [41]–[43].

These considerations, together with the results of our directed evolution experiments, lead to the hypothesis that probable radiation-induced oxidative damage has been one of the driving forces in the evolution of cyanobacterial/eukaryotic L/D1 proteins.

The results presented in this paper strengthen the role of the photosynthetic reaction centre D1 protein in the tolerance/resistance to extreme environmental conditions. We demonstrate that even single amino acid substitutions in D1 enable the cells to survive in the presence of free radicals produced by both high light fluencies and ionizing radiation. Further, the *in silico* analysis of L/D1 amino acid sequences indicates that apparently radiation-induced oxidative damage has been one of the driving forces in the evolution of these proteins and gives evidence of a molecular signature of different levels of ionizing radiation on Earth during the evolution of the photosynthetic apparatus. From a methodological point of view, the experimental approach presented in this paper is suitable for identifying molecular adaptations to challenging environmental conditions. This approach allows the selection of mutants with improved stability, a parameter of great interest in any biotechnological application of photosynthetic proteins spanning from space research to biosensoric and photovoltaic fields, including biofuel and nutraceuticals production [44].

## Materials and Methods

### Strains, Growth Conditions and Media

Functional characterization of the reference strain and the D1 mutants was carried on cell cultures in the early exponential growth phase (OD_750_ = 0.4) containing about (0.8±0.1)×10^6^ cells per ml. The cultures were grown on Tris-acetate-phosphate (TAP) [45] medium under continuous illumination of 50 µmol photons m^−2^ s^−1^, 25°C and agitation at 150 rpm.

The main chlamydomonas strains exploited were IL, a mutant containing an intronless *psb*A gene [46] that represented the control reference strain for characterization of the D1 mutants, and Del1, a mutant used as a recipient strain for D1 mutants generation. The latter is a derivative of the IL reference strain [24] lacking an approx 0.4 kb fragment encoding amino acids Ala153 to Ala294 of the PSII D1 protein. As a result of the deletion the strain synthesizes a truncated D1 protein and is able to grow only mixotrophically on TAP medium, using acetate as a carbon source. Because of this, the acetate-free high-salt (HS) medium was used as a selection pressure for photoaoutotrophic growth. It has been previously shown that the photoautotrophically incompetent Del1 strain can be transformed with PCR-generated *psb*A fragments, resulting in colonies able to grow photoautotrophically on HS medium [47]. When necessary, chlamydomonas strains were grown in HS and TAP media, solidified with 1.5% agar.

### Random and site-directed PCR-based mutagenesis

Random and site-directed mutageneses were performed by PCR using as a template the pSH5 plasmid, harboring the intronless *psb*A gene. The resulting DNA fragments of the *psb*A gene were used for chlamydomonas chloroplast transformation without further purification steps and cloning procedures, as previously reported [47].

#### Directed evolution experiment to isolate radiation tolerant D1 mutant strains

Random mutagenesis was carried out using a commercially available kit (BD Biosciences Clontech, Palo Alto, CA, US) to generate pools of random mutated *psb*A sequences by error-prone PCR. In particular, 50 µl reaction mixture containing 1 ng plasmid DNA and 25 pmol of each primer were utilized, under buffer conditions 4 and 6 of the kit, corresponding to mutation frequencies of 3.5 and 4.8 mutations/kbp, respectively. The products of the mutagenic reactions, of approx. 0.7 kbp length, were quantified and analyzed by agarose gel electrophoresis and ethidium bromide staining.

Chlamydomonas transformation was carried out on Del1 cultures grown in liquid TAP medium up to mid-log phase and 2×10^7^ cells. Algal cells were concentrated onto nitrocellulose filters (0.45 µm, 47 mm in diameter, NL17, Whatman GmbH, Dassel, Germany) and placed on TAP agar medium overnight in the dark. The filters were bombarded with around 1 µg of the error-prone PCR product precipitated onto tungsten particles [48]. A homemade particle gun with helium pressure (13 bar pressure for acceleration; 12 cm shooting distance; 1.8 bar vacuum) was used similar to the device described in [49]. The bombarded filters were kept on TAP agar medium in the dark overnight and then transferred to the selective HS agar medium. The successfully transformed cells grow photoautotrophically and form single colonies usually after 10–14 days in continuous light (50 µmol m^−2^ s^−1^).

#### Irradiation exposures and neutron and proton facilities

For the irradiation experiments, samples were prepared as described in the following. Single colonies were picked up from the filters, transferred into a liquid HS medium and grown for 3 weeks. The resulting liquid culture contained D1 random mutants as well as cells of the reference strain IL that were produced by recombination events of Del1 with non-mutated *psb*A gene fragments occurring in the error-prone PCR reaction. Around 1.5×10^7^ cells in the mid-log growth phase of the mixture were spotted in the centre of plates with HS agar -medium and exposed to the different irradiation sources.

The experiments with fast neutrons (800 MeV) were performed on one of the secondary beam lines installed on the Super Proton Synchrotron in CERN (Conseil Européen pour la Recherche Nucléaire, Meyrin, Switzerland) as described in [50].

The experiments with neutrons and neutron plus high light fluency rate (500 µmol m^−2^ s^−1^) were performed at the Frascati Neutron Generator (FNG) laboratory of the Italian National Agency for New Technologies, Energy and Sustainable Economic Development. The FNG uses a deuteron beam accelerated up to 300 keV impinging on a source to produce a 14 MeV neutron output via the T(d,n) α fusion reaction. The exposure experiment with protons was performed at the South National Laboratory of the National Institute for Nuclear Physics in Catania, Italy using one of the beam lines of the Tandem accelerator. The protons used in the experiment had the highest energy (27 MeV) the accelerator could provide. More details about irradiation experiments are presented in Figure S3.

About 2000 chlamydomonas D1 random mutants were exposed to both neutron and proton sources.

After the exposure, plates were kept under continuous light until single colonies became visible (after around 2 weeks). The single colonies of the survivors were selected, grown on HS liquid medium and analysed by *psb*A gene sequencing.

As a whole, neutron exposures allowed the selection of 14 transformed colonies. Nucleotide sequence analyses indicated that among them, 10 strains contained different single or double mutations in the *psb*A gene, and 4 carried the naïve gene. A large number of cells survived the proton exposures and 18 of them were analyzed by *psb*A gene sequencing, revealing single, double, triple mutations and naïve genes (8 colonies).

#### Site-directed mutagenesis

Site-directed mutagenesis was performed using *psb*A fragments produced in two-steps PCR. In the first step, two DNA fragments, upstream and downstream from the position of the target point mutation were synthesized (for example in the case of I163T mutants the one corresponding to position 163 in the D1 protein). Using the I163T mutant as an example for the synthesis of the upstream fragment, an outer forward primer and 163-reverse primer, which starts at the codon in the *psb*A gene that corresponds to position 162 in the D1 protein, were used. Similarly, for the downstream fragment a 163-forward primer, which starts at the codon in the *psb*A gene that corresponds to position 164 in the D1 protein and an outer reverse primer were utilized. The sequences of the primers exploited for the site-directed mutants generation are reported in Table S2. PCR amplicons were analyzed by agarose gel electrophoresis, extracted and purified using the commercial kit SV Gel and PCR Clean-Up System (Promega, USA). The purified DNA fragments were used in the second PCR in the presence of the two mutagenic primers, hosting the target mutation, together with the outer primers from the first PCR (Table S2). The fragments obtained from the second PCR were analyzed and the amplicon of interest was purified as described above and amplified in subsequent standard PCR. This mutated amplicon was directly used for the algal biolistic transformation as described above. A single colony was picked from a bombarded filter and was transferred to HS liquid medium. The point mutation was verified by *psb*A gene sequencing (Seqlab, Göttingen, Germany). The homoplasmicity of the *psb*A mutant was verified by standard PCR and agarose gel electrophoresis. Only homoplasmic colonies were used in all the described experiments.

### Functional characterization of the site-directed mutant strains

The mixotrophic growth rate was estimated by measuring the absorption of the culture at 750 nm, which is proportional to the total cell number, and the total chlorophyll content spectrophotometrically (Perkin-Elmer Lambda 40 UV/VIS, Norwalk CT, USA) for a period of 88 h under growth conditions.

The oxygen evolution capacity of the selected strains was measured on TAP liquid cell cultures, containing 15±2 mg l^−1^ chlorophyll, at 24°C using a Clark-type oxygen electrode (Chlorolab 2, Hansatech, Instr. Ltd, Norfolk, UK) in the presence of 10 mM NaHCO_3_ [51] as an additional carbon source. Samples were illuminated with increasing light intensities provided by red LEDs (660 nm) under continuous stirring. The rate of oxygen evolution under each of the light conditions was recorded continuously for a period of 2 min.

The chlorophyll fluorescence induction curves were registered on 10 min dark-adapted liquid cell cultures grown in TAP medium, containing 14±1 mg l^−1^ chlorophyll, by Plant Efficiency Analyzer (PEA, Hansatech Instr. Ltd, Kings Lynn, Norfolk, UK). The excitation light, a 3 sec saturated pulse (intensity of 600 W/m^2^), was provided by an array of six red light-emitting diodes (peak at 650 nm), focused on the surface of the sample. The maximum quantum yield of PSII photochemistry is calculated as Fv/Fm = (Fm -F_50µs_)/Fm and the efficiency of the electron transport between the primary (Q_A_) and the secondary (Q_B_) PSII electron acceptors as 1-V_J_ where 1-V_J_ = 1-(F_2ms_-F_50µs_)/(Fm -F_50µs_), and F_50µs_, Fm and F_2m_ are the initial fluorescence, the maximum fluorescence and the fluorescence level 2 ms after the beginning of the illumination, respectively [52].

### Analyses of photosynthesis-damaging light conditions

The photosynthetic performance of the site-directed mutants was determined by chlorophyll fluorescence analyses, using the Photo II device (Biosensor Srl, Palombara Sabina, RM, Italy, http://www.biosensor.it/). The fluorescence induction curves were recorded every hour, using a 6 s pulse provided by four red LEDs. In addition, the instrument is designed to provide the white light necessary to maintain the photosynthetic reaction of the samples. Cell cultures in early exponential growth phase, containing about 80 µg total chlorophyll, were harvested by weak centrifugation and layered on TAP agar medium in the instrument containers. The intensity of the photosynthetic actinic light was set at 50 and 150 µmol m^−2^ s^−1^ and the maximum quantum yield of PSII photochemistry was followed for a period of 7 days. Previous experiments and results in this paper indicated that 150 µmol m^−2^ s^−1^ were stressful for chlamydomonas cells in immobilized conditions (Rea G, unpublished data; Fig. 4).

### Statistical analyses

The presented data are means of 6–9 values obtained in three independent experiments. A nonparametric equivalent to analysis of variance was used (Kruskal-Wallis ANOVA) to define the differences between the reference strain and the mutants. In the comparative analysis of the amino acid composition of eukaryotic, cyanobacterial and bacterial D1/L proteins 50 aminoacidic sequences were aligned. For the overall mean comparison, analysis of variance (one way ANOVA) was applied. The statistical significance of the differences was evaluated by p-level.

### Analysis of the amino acid composition of bacterial L and eukaryotic and cyanobacterial D1 proteins

In this paper, general properties of the D1 amino acid substitutions were described (Table S1) in terms of side chain polarity [53], hydropathy index [54] and reactivity classes, as reported in Table 2. The clustering was done according to the reactivity scale deduced from the studies reviewed in [30], which demonstrated that polar residues are less prone to damage by reactive oxygen species while reactivity of the aliphatic amino acids increases with the number of methyl groups. Sulphur containing and aromatic amino acids are, in the reported order, the most prone to oxidative damage (Table 2).

**Table 2:** Amino acid classification according to increasing reactivity towards reactive oxygen species.

Class of reactivity^a^	0	I	II	III	IV	V	VI
Amino acid	Polar residues^b^	Gly	Ala	Pro	Ile	Met	Trp
				Val	Leu	Cys	Phe
							Tyr

a The clustering was done according to [30].

b Excluding sulfur-containing and aromatic polar amino acid residues.

*R. sphaeroides* L and *C. reinhardtii* D1 whole-length amino acid sequences were used as baits in a BLAST [55] search of the NCBI non-redundant protein sequences database. Significant hits were retrieved and sequences with an identity percentage >90% were removed. Average amino acid composition and standard deviation were then calculated for a dataset of 50 sequences using the Perl program freqaa.pl [56] and the GraphPad Prism statistical analysis tools.

For the composition analysis of the Q_B_ binding niche, the *R. sphaeroides* L protein 174–249 region was selected, encompassing three α helices and two loops which build up the niche. The corresponding region of *C. reinhardtii* D1 protein (residues 199–292) was selected by structure-based sequence alignment obtained by superimposing the three dimensional structures of *R. sphaeroides* L (PDB code 2BOZ) [57] and *Termosynechococcus elongatus* D1 (PDB code 2AXT) [21].

Compositional analysis of cyanobacterial proteins was carried out using the *Chlamydomonas reinhardtii* D1 whole-length amino acid sequence as bait in a BLAST search of the cyanobacterial subset of the NCBI non redundant protein sequences database. As for bacterial and eukaryotic proteins, significant hits were retrieved and sequences with an identity percentage >90% were removed before average amino acid composition calculation.

## Supporting Information

Figure S1 **Characterization of the mixotrophic growth rate of site-directed chlamydomonas D1 mutants.** The time courses of cell culture growth (A) and chlorophyll accumulation (B) expressed as a ratio between the chlorophyll content and the optical density at 750 nm were carried out for a period of 88 h under growth conditions. The difference between the mutants and the reference strain gradually increased with time. Values are the average of four independent experiments, ±SE, n = 4. The chlorophyll content of the mutants was statistically different from the reference strain at P = 0.05.
(TIF)

Figure S2 **Structural superimposition of** ***Termosynechococcus elongatus*** **D1 and** ***Rodobacter spheroides*** **L reaction centre proteins.** Structure of *T. elongatus* D1 protein (PDB code 2AXT) [21] and *R. spheroides* L protein (PDB code 2BOZ) [57] were retrieved and homology modelled. Superimposition reveals a backbone fold highly compatible in the 190–291 region (approx 25% identity). D1 backbone is coloured in dark blue, L backbone is coloured according to root mean square deviation values: warm colours indicate higher deviations (the deviation in loop regions is depicted in red); blue arrows indicate the D1 region hosting the Q_B_ binding pocket (aa 199–292).
(TIF)

Figure S3 **Directed evolution strategy to isolate radiation tolerant chlamydomonas D1 mutants.** The FNG deuteron beam produces a nearly isotropic neutron flux; it assumes the same value over a spherical surface area of radius r centred on the source and its intensity decreases as r^−2^. Considering the geometry of the neutron beam, an optimised holder was built to expose several samples at the same time to two different doses (35 and 75 mGy) in the presence or absence of high light intensity. For each of the several experiments performed, the absorbed neutron doses were evaluated using the Monte Carlo N-Particle transport code with an uncertainty of 5%. The tandem proton accelerator in Catania provides a collimated cylindrical shaped beam, which results in a homogeneous inner circular surface area with a diameter of about 2 cm. For this reason, for the exposure, algal cells were distributed on a surface area <2 cm diameter. Such a preparation allowed the biological component to absorb a uniform dose. Each sample was directly exposed in air, perpendicularly to the beam. During the experiment two different doses were provided, about 0.5 and 5 Gy, measured using an ionization chamber interposed between the exit point of the beam and the sample. The doses were delivered to each sample during an exposition time of about 6 seconds.
(TIF)

Table S1 Description of amino acid substitutions in *C. reinhardtii* D1 random mutants surviving after irradiation exposures.
(DOC)

Table S2 Sequences of primers used in the two-step PCR for the site-directed mutagenesis.
(DOC)
