# Supplementary figures and images for: Directed Evolution and In Silico Analysis of Reaction Centre Proteins Reveal Molecular Signatures of Photosynthesis Adaptation to Radiation Pressure
Source: PLoS One. 2011 Jan 13;6(1):e16216. doi: 10.1371/journal.pone.0016216 (PMC3020971; doi:10.1371/journal.pone.0016216)

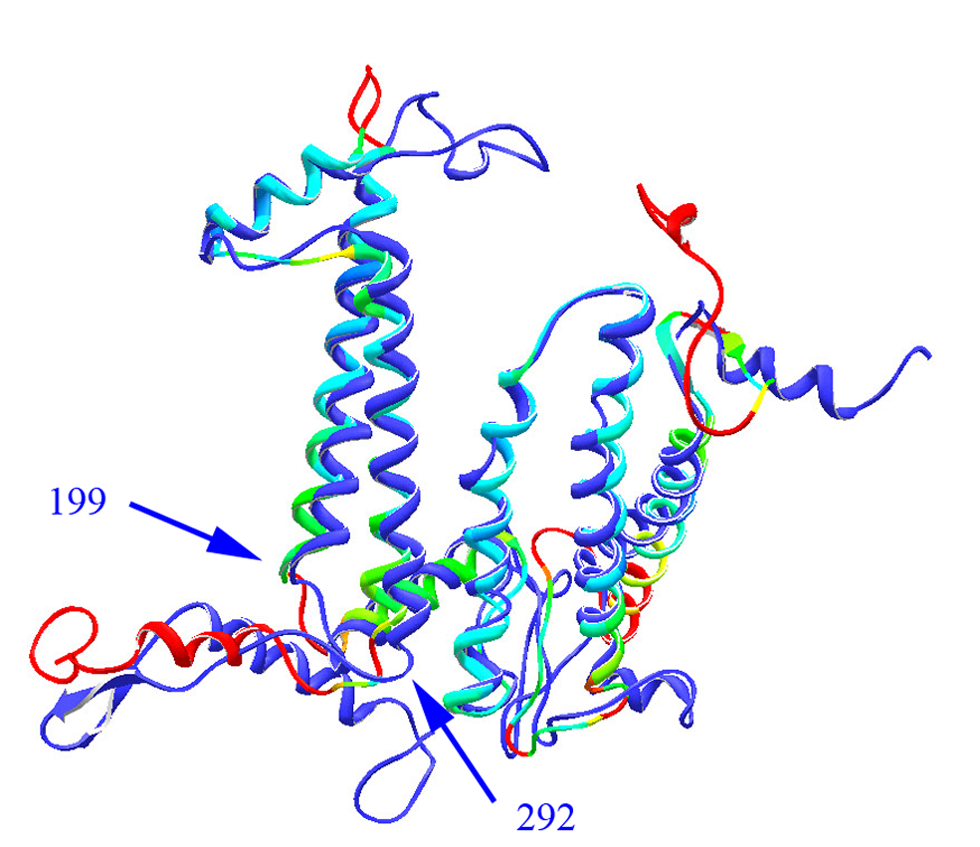

Supplement: Figure S2 — Structural superimposition of Termosynechococcus elongatus D1 and Rodobacter spheroides L reaction centre proteins. Structure of T. elongatus D1 protein (PDB code 2AXT) [21] and R. spheroides L protein (PDB code 2BOZ) [57] were retrieved and homology modelled. Superimposition reveals a backbone fold highly compatible in the 190–291 region (approx 25% identity). D1 backbone is coloured in dark blue, L backbone is coloured according to root mean square deviation values: warm colours indicate higher deviations (the deviation in loop regions is depicted in red); blue arrows indicate the D1 region hosting the QB binding pocket (aa 199–292). (TIF) [file pone.0016216.s002.tif]

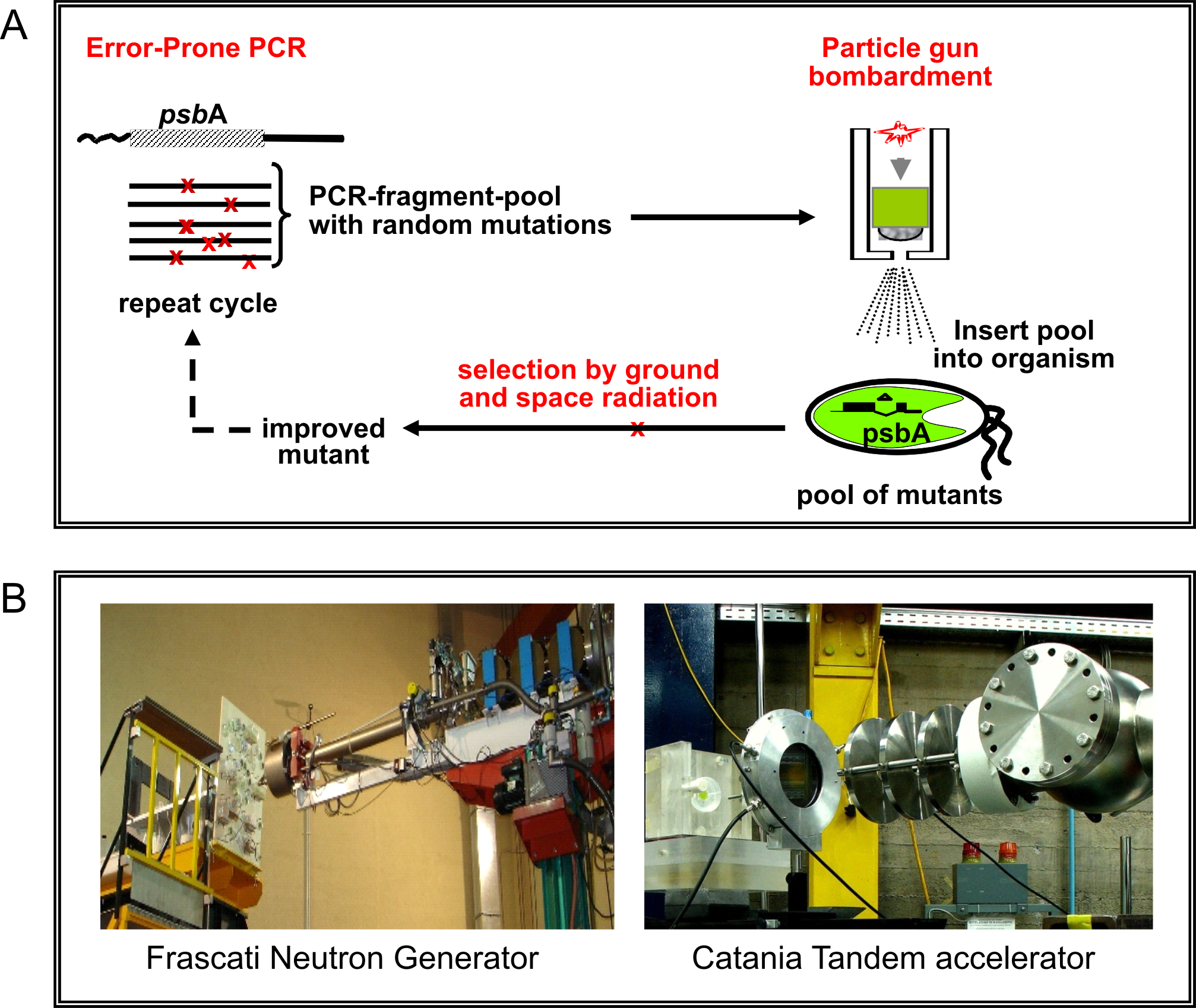

Supplement: Figure S3 — Directed evolution strategy to isolate radiation tolerant chlamydomonas D1 mutants. The FNG deuteron beam produces a nearly isotropic neutron flux; it assumes the same value over a spherical surface area of radius r centred on the source and its intensity decreases as r−2. Considering the geometry of the neutron beam, an optimised holder was built to expose several samples at the same time to two different doses (35 and 75 mGy) in the presence or absence of high light intensity. For each of the several experiments performed, the absorbed neutron doses were evaluated using the Monte Carlo N-Particle transport code with an uncertainty of 5%. The tandem proton accelerator in Catania provides a collimated cylindrical shaped beam, which results in a homogeneous inner circular surface area with a diameter of about 2 cm. For this reason, for the exposure, algal cells were distributed on a surface area <2 cm diameter. Such a preparation allowed the biological component to absorb a uniform dose. Each sample was directly exposed in air, perpendicularly to the beam. During the experiment two different doses were provided, about 0.5 and 5 Gy, measured using an ionization chamber interposed between the exit point of the beam and the sample. The doses were delivered to each sample during an exposition time of about 6 seconds. (TIF) [file pone.0016216.s003.tif]
