# Supplementary material for: Directed Evolution and In Silico Analysis of Reaction Centre Proteins Reveal Molecular Signatures of Photosynthesis Adaptation to Radiation Pressure
Source: PLoS One. 2011 Jan 13;6(1):e16216. doi: 10.1371/journal.pone.0016216 (PMC3020971; doi:10.1371/journal.pone.0016216)
Supplement: Table S1 — Description of amino acid substitutions in C. reinhardtii D1 random mutants surviving after irradiation exposures. (DOC) [file pone.0016216.s004.doc]

**Table S1.** Description of amino acid substitutions in *C. reinhardtii* D1 random mutants surviving after irradiation exposures.

| Mutants | Amino acid  substitutions | | | | Amino acid properties  hydropathy index^a^/reactivity class/side chain polarity | | | | | | Localization of the mutation in the protein |
| --- | --- | --- | --- | --- | --- | --- | --- | --- | --- | --- | --- |
|  | wild type → mutated | | | | wild type → mutated | | | | | |  |
| *Mutants selected under high energy neutrons* | | | | | | | | | | | |
| V160A | valine | | | alanine | | 4.2 (III) | nonpolar | 1.8 (II) | nonpolar | | near to Tyr_161_­ d(Cα-Cα) = 3.8 Å |
| I163N | isoleucine | | | asparagine | | 4.5 (IV) | nonpolar | -3.5 (0) | polar | | near to Tyr_161_­ d(Cα-Cα) = 5.6 Å |
| L159I/N230Y | leucine | | | isoleucine | | 3.8 (IV) | nonpolar | 4.5 (IV) | nonpolar | | near to Tyr_161_­ d(Cα-Cα) = 5.9 Å |
|  | asparagine | | | tyrosine | | -4.5 (0) | polar | -1.3 (IV) | polar | | in the loop between IV and V helix of D1 |
| *Mutants selected under neutrons* | | | | | | | | | | | |
| L159I/I184V | | leucine | | isoleucine | | 3.8 (IV) | nonpolar | 4.5 (IV) | nonpolar | near to Tyr_161_­ d(Cα-Cα) = 5.9 Å | |
|  | | isoleucine | | valine | | 4.5 (IV) | nonpolar | 4.2 (III) | nonpolar | near to OEC d(Cα-Mn4) = 11.1 Å | |
| F197L/F285L | | phenylalanine | | leucine | | 2.8 (VI) | nonpolar | 3.8 (IV) | nonpolar | in the helix IV of D1 | |
|  | | phenylalanine | | leucine | | 2.8 (VI) | nonpolar | 3.8 (IV) | nonpolar | in the helix V of D1 | |
| G207S | | glycine | | serine | | -0.4 (I) | nonpolar | -0.8 (0) | polar | in the helix IV of D1 | |
| *Mutants selected under neutrons plus high light* | | | | | | | | | | | |
| L159M | | leucine | | methionine | | 3.8 (IV) | nonpolar | 1.9 (V) | nonpolar | | near to Tyr_161_­ d(Cα-Cα) = 5.9 Å |
| S177P | | serine | | proline | | -0.8 (0) | polar | -1.6 (III) | nonpolar | | near to OEC d(Cα-Mn4) = 14.3 Å |
| I163T | | isoleucine | | threonine | | 4.5 (IV) | nonpolar | -0.7 (0) | polar | | near to Tyr_161_­ d(Cα-Cα) = 5.6 Å |
| L200I | | leucine | | isoleucine | | 3.8 (IV) | nonpolar | 4.5 (IV) | nonpolar | | in the helix IV of D1 |
| *Mutants selected under protons* | | | | | | | | | | | |
| F158Y/I184N/N230D | | | phenylalanine | tyrosine | | 2.8 (VI) | nonpolar | -1.3 (VI) | polar | | near to Tyr161­ d(Cα-Cα) = 6.7 Å |
|  | | | isoleucine | asparagine | | 4.5 (IV) | nonpolar | -3.5 (0) | polar | | near to OEC d(Cα-Mn4) = 11.1 Å |
|  | | | asparagine | aspartic acid | | -3.5 (0) | polar | -3.5 (0) | polar | | in the loop between IV and V helix of D1 |
| P162S | | | proline | serine | | -1.6 (III) | nonpolar | -0.8 (0) | polar | | near to Tyr_161_­ d(Cα-Cα) = 3.9 Å |
| S268P/M293T | | | serine | proline | | -0.8 (0) | polar | -1.6 (III) | nonpolar | | in the loop between IV and V helix of D1 |
|  | | | methionine | threonine | | 1.9 (V) | nonpolar | -0.7 (0) | polar | | in the helix V of D1 |
| M172T | | | methionine | threonine | | 1.9 (V) | nonpolar | -0.7 (0) | polar | | near to OEC d(Cα-Mn4) = 9.1 Å |
| M172L | | | methionine | leucine | | 1.9 (V) | nonpolar | 3.8 (IV) | nonpolar | | near to OEC d(Cα-Mn4) = 9.1 Å |
| M183T | | | methionine | threonine | | 1.9 (V) | nonpolar | -0.7 (0) | polar | | near to OEC d(Cα-Mn4) = 10.9 Å |
| L210I | | | leucine | isoleucine | | 3.8 (IV) | nonpolar | 4.5 (IV) | nonpolar | | in the helix IV of D1 |
| I224F/E226V | | | isoleucine | phenylalanine | | 4.5 (IV) | nonpolar | 2.8 (VI) | nonpolar | | in the helix IV of D1 |
|  | | | glutamic acid | valine | | -3.5 (0) | polar | 4.2 (III) | nonpolar | | in the loop between IV and V helix of D1 |
| F274Y | | | phenylalanine | tyrosine | | 2.8 (VI) | nonpolar | -1.3 (VI) | polar | | in the helix V of D1 |
| I281T | | | isoleucine | threonine | | 4.5 (IV) | nonpolar | -0.7 (0) | polar | | in the helix V of D1 |

^a^The irradation involved exposures to high energy neutrons (800 MeV), neutrons (14 MeV), neutrons (14 MeV) plus high light (500 µmol m^-2^ s^-1^), or protons (27 MeV) in different on ground facilities. General view of the amino acid substitutions identified in the deduced protein primary structure by sequence analyses of the *psb*A gene encoding the D1 protein in the survived strains. ^a^ The side chain polarity is according to [53], the hydropathy index is cited as in [54], the amino acids reactivity classes are reported in Table 2

.
