# Supplementary material for: Directed Evolution and In Silico Analysis of Reaction Centre Proteins Reveal Molecular Signatures of Photosynthesis Adaptation to Radiation Pressure
Source: PLoS One. 2011 Jan 13;6(1):e16216. doi: 10.1371/journal.pone.0016216 (PMC3020971; doi:10.1371/journal.pone.0016216)
Supplement: Table S2 — Sequences of primers used in the two-step PCR for the site-directed mutagenesis. (DOC) [file pone.0016216.s005.doc]

**Table S2.** Sequences of primers used in the two-step PCR for the site-directed mutagenesis.

| **1^st^ PCR** | | **2^nd^ PCR** | |
| --- | --- | --- | --- |
| **Primers** | **Sequence (5’ → 3’)** | **Primers^a^** | **Sequence (5’ → 3’)** |
| **outer for** | GGTGCTGTAATCCCAACTTCT |  |  |
| **outer rev** | CTAGAGTTAGTTGAAGCTAAGTCTAGAGGGA |  |  |
| **I163 rev** | AGGGTAAACTAAGAATACAGC | **I163Tmut-rev** | CTTAGTTTACCCT**ACC**GGCCAAGGTTCATTCTCTG |
| **I163 for** | GGCCAAGGTTCATTCTCTG | **I163Tmut-for** | GAACCTTGGCC**GGT**AGGGTAAACTAAGAATACAGC |
| **P162rev** | GTAAACTAAGAATACAGCTGAAGC | **P162S mut rev** | GAACCTTGGCCGAT**TGA**GTAAACTAAGAATACAGCTGAAGC |
| **P162for** | ATCGGCCAAGGTTCATTCTC | **P162S mut for** | CTGTATTCTTAGTTTAC**TCA**ATCGGCCAAGGTTCATTCTC |
| **L200rev** | CATGTGGAATGGGTGCATAAG | **L200I mut rev** | CACCAGCAACACC**AAT**CATGTGGAATGGGTGCATAAG |
| **L200for** | GGTGTTGCTGGTGTATTCG | **L200I mut for** | CACCCATTCCACATG**ATT**GGTGTTGCTGGTGTATTCG |
| **I281rev** | TACCGGCCAAGCAGCTAAG | **I281T mut rev** | GTGAACCAAATACC**TGT**TACCGGCCAAGCAGCTAAG |
| **I281for** | GGTATTTGGTTCACTGCTTTAG | **I281T mut for** | GCTGCTTGGCCGGTA**ACA**GGTATTTGGTTCACTGCTTTAG |
| **G207rev** | GAATACACCAGCAACACCTAAC | **G207S mut rev** | CTGAGAATAATGAACC**TGA**GAATACACCAGCAACACCTAAC |
| **G207for** | GGTTCATTATTCTCAGCTATGC | **G207S mut for** | GTGTTGCTGGTGTATTC**TCA**GGTTCATTATTCTCAGCTATGC |
| **M172rev** | ACCGTCAGAGAATGAACCTTGG | **M172L mut rev** | CAGAGATACCTAAAGG**TAA**ACCGTCAGAGAATGAAC |
| **M172for** | CCTTTAGGTATCTCTGGTAC | **M172L mut for** | GTTCATTCTCTGACGGT**TTA**CCTTTAGGTATCTCTG |

^a^The modified nucleotides in the mutagenic primers are indicated.
